# Emergency Medicine Residency Website Wellness Pages: A Content Analysis

**DOI:** 10.5811/westjem.34873

**Published:** 2025-05-16

**Authors:** Alexandra Sappington, Brian Milman

**Affiliations:** * Louisiana State University Health Sciences Center, Department of Emergency Medicine, New Orleans, Louisiana; † University of Texas Southwestern Medical Center, Department of Emergency Medicine, Dallas, Texas

## Abstract

**Introduction:**

The COVID-19 pandemic impacted the way medical students seek residency positions. In 2020, the Accreditation Council for Graduate Medical Education advocated for virtual interviews. Most emergency medicine (EM) interviews in 2023 remained virtual, and this format will persist for the foreseeable future. Since students are not evaluating programs in person in most cases, residency websites are crucial for prospective residents. Resident wellness is critical for resident training and important to prospective residents; it follows that programs must be transparent about resident wellness on websites. In this study we aimed to quantify the number of EM programs with wellness pages on their websites and identify themes portrayed on those pages.

**Methods:**

We analyzed residency website wellness pages from EM websites based on the 2022 directory of the Electronic Residency Application Service. We independently coded wellness statements through an inductive process. Codes were revised iteratively to consensus and organized into themes.

**Results:**

We identified 278 (100%) EM residency websites. Of these websites, 57 (20.5%) had a wellness page, 45 (16.2%) linked to an institutional page that discussed wellness, 169 (60.8%) discussed wellness themes on their website in areas other than a wellness page, and 69 (24.8%) had no direct mention of wellness anywhere on their website. Using this information, we identified themes including community involvement, growth and development, nutrition and health, psychological well-being, social and relaxation activities, wellness culture and environment, wellness curriculum, wellness structure and resources, and work-life integration.

**Conclusion:**

Most EM program websites do not include a wellness page. Of the programs that do, we identified important themes. The absence of dedicated wellness pages on most EM websites suggests an opportunity for programs to better communicate their wellness initiatives to applicants, helping them identify programs that align with their values.

## INTRODUCTION

Physician wellness is critical.^1^ Burnout, as defined in the 1970s by Herbert Freudenberger, details the repercussions of significant amounts of stress in “helping professions.”^2^ Emergency medicine (EM) has a higher rate of burnout when compared to other specialties.^3^ A national EM resident wellness survey study disseminated in 2017 found that 77.7% of residents were identified as burned out.^4^ The COVID-19 pandemic exacerbated levels of burnout in subsequent years.^3^

The COVID-19 pandemic also created a shift in the way medical students apply for residency. Specifically, in June 2020 the Accreditation Council for Graduate Medical Education released a statement that advocated for virtual interviews.^5^ The Association of American Medical Colleges advocated for a continuation of virtual interviews for the 2022–2023 cycle, as it eliminates financial and scheduling challenges for programs and applicants. The Council of Residency Directors in Emergency Medicine further encouraged EM residency programs to follow virtual interview guidelines. It appears that virtual residency interviews will remain the dominant format for years to come. Emergency medicine-bound students list program websites as the most important factor in determining their rank lists in the post-COVID-19 era and rank program websites as important in determining faculty reputation, program diversity and inclusion, and program culture.^6^,^7^ We can expect EM students to continue to rely heavily on program websites for the foreseeable future.

Residency training is known to have negative effects on physical, emotional, and social wellbeing.^8^ Eliminating the in-person evaluation of perceived happiness and comradery among residents will require programs to be transparent about resident wellness on websites.^5^ A survey conducted in 2022 showed that resident wellness was identified by medical students as the most important content on a residency website.^9^ Studies have been performed evaluating wellness content on program websites in several fields, but not in EM. By analyzing how wellness is presented on EM websites, we aimed to provide insight into the current landscape of this aspect of wellness communication.

## METHODS

We obtained a list of all EM residency programs accepting Electronic Residency Application Service (ERAS) applications for the 2022 application cycle. The EM ERAS directory was accessed in March 2023, and we accessed each EM program website between April 1–April 30, 2023. If the ERAS directory did not link to a program website, we performed a Google search to identify the residency program website. Each program website was reviewed by a single reviewer to determine whether there was a wellness page on the website. We also reviewed each website in its entirety to determine whether other pages on the website discussed themes of wellness or wellbeing. If a website linked to an institutional page discussing wellness separate from the residency website, that page was reviewed as well. Wellness pages and linked graduate medical education (GME) pages were recorded in an Excel spreadsheet with the full text from that webpage.

At the time of analysis, AS was a medical student, bringing a unique perspective as a prospective applicant navigating the residency application process. BM was an associate program director during analysis. The combination of these viewpoints allowed for a more comprehensive understanding of the wellness information presented on residency program websites. Using constructivist grounded theory, each author independently examined the full text from 15 wellness pages to generate initial codes. AS identified 138 descriptive codes, and BM identified 70 descriptive codes (184 unique codes combined). After discussion and review of each of the first 15 statements, areas of overlap were identified, and the initial codes were consolidated into a codebook containing 47 codes.

**Population Health Research Capsule**

What do we already know about this issue?

*Wellness is important to applicants when deciding how to rank residency programs. However, wellness content is not always available on program websites.*

What was the research question?

*What percentage of EM programs have a wellness page on their website and what themes are discussed?*

What was the major finding of the study?

*20.5% of programs had a wellness page, while 24.8% did not mention wellness anywhere on their website.*

How does this improve population health?

*Enhancing website wellness content can improve applicant decision-making and encourage programs to think deliberatively about their wellness initiatives.*

We independently reviewed an additional 10 website texts, and the codebook was revised. One code was added, two were removed, and five were redefined, resulting in 46 final codes. We then re-coded the first 25 wellness statements using the updated codebook. The coding structure was stable, and no additional codes were added to the codebook. We coded the remaining wellness statements and identified themes. We then held a Zoom meeting to discuss and resolve discrepancies. During this meeting, we reviewed the source text simultaneously with the codebook open. Through discussion and mutual agreement, we resolved all discrepancies without having to involve an additional coder or arbitrator. Themes were identified by grouping related codes into broader conceptual categories that represented patterns in website wellness content. We discussed these themes and agreed upon them. This study was determined to be non-regulated research by the University of Oklahoma Internal Review Board in February 2023.

## RESULTS

We identified 278 EM residency programs based on the 2022 ERAS Directory list of participating programs and specialties. Websites were identified and accessed for 278 (100%) programs. Fifty-seven programs (20.5%) had a main page or subpage dedicated to wellness or wellbeing, 169 (60.79%) programs discussed wellness somewhere on their website other than on a page dedicated to wellness, 45 (16.19%) programs linked to a GME page highlighting wellness, and 69 (24.82%) programs did not directly mention wellness or wellbeing anywhere on their website. Programs were counted in multiple categories if information was included in multiple areas of their website.

Of the 57 programs that had a page dedicated to wellness on their departmental website, 22 (38.6%) were titled “Wellness,” 12 (21.05%) were titled “Resident Wellness,” and the remainder were a variation of wellness or wellbeing. A complete list of page titles can be found in Appendix A. One wellness page contained pictures only, and it was not included in the content analysis. The most common subjects discussed on wellness pages included social events, mental health, physical health, institutional support, wellness didactics, and burnout. The percentage of programs that discussed each subject can be found in Table 1. The least common subjects, defined as <5%, that appeared on residency wellness pages were empathy, achievement, personal development, legal concerns, leadership skill development, lack of personal fulfillment, imposter syndrome, and harassment.

Nine broader themes emerged from analysis of EM residency website wellness pages:

### Theme 1: Social and Relaxation Activities

The most common theme that appeared was social and relaxation activities. 83.9% of programs highlighted retreats, class activities, and other social events on their wellness page and 68.4% included pictures of their residents participating in social activities.

These outings include paintball, large group dinners, and outdoor activities such as skiing and team sports. In the past they have organized softball games and ping-pong and bowling tournaments between the other local EM residencies.

### Theme 2: Psychological Wellbeing

Many programs found it difficult to discuss wellness without discussing burnout. Programs also included their approach to mitigating burnout and building resilience.

Healthcare providers are not immune to poor wellness and well-being, and their high prevalence of burnout, depression, anxiety, and sleep disorders are all contributing factors.

At this monthly get together, at an attending’s house we discuss building resilience and professional excellence and externalizing and highlighting serious threats to wellness like substance abuse, interpersonal conflict, and PTSD.

Programs acknowledged that residents are partners in improving wellness and the best initiatives are often resident driven.

We understood the importance of resident input and feedback into their own wellness. Who else would know what residents need, in terms of wellness, other than residents themselves?

This year we added a 4th elected chief resident position specifically dedicated to wellness!

Wellness is not one size fits all and frequently requires a more individualized approach.

We understand that wellness is not mandatory events, meditation, and yoga for everyone. While we have a robust curriculum to explore the different avenues of wellness, we encourage our residents to identify their own stress relieving practices and to maintain those activities to avoid burning out.

While we know that residency is hard, we also know that “wellness” is a moving target and that which makes a person “well” is highly individualized.

### Theme 3: Nutrition and Health

About half of programs mentioned food available to residents while at the hospital and on shift, as well as gyms and other physical fitness resources available to residents.

Residents have their own dedicated lounge and fully stocked fridge with food and drinks. There are food trucks available at nearly all hours of the night out front of the main facility to take care of those evening and late-night cravings. Tired of the cafeteria food? Need something quick? The snack shack in the main ED provides this – of course available to use with your meal stipend.

### Theme 4: Wellness Structure and Resources

Many programs had both departmental and institutional structure to wellness and addressed their holistic approach on their website.

The infrastructure supports and promotes preventative care, healthy living, mental health, second-victim support, work-life balance, and peer-peer counseling and mentoring.

Our wellness activities focus on service, resiliency, and career development, and will continue to grow with creative ideas to support and empower residents.

Through intentional reflective practices, didactic sessions, and interactive social opportunities, our goal is to help residents maintain perspective and create healthy habits that promote longevity in Emergency Medicine.

Programs also included information about counseling services or methods of monitoring mental health throughout residency.

Trainees are required to complete the Well-being Index twice each year while in their training program. During resident/fellow semi-annual review meetings with their Program Director, one of the topics for discussion will be the trainee’s self-care and completion of the Well-being Index.

Additionally, 42.9% of wellness pages talked about a wellness committee.

Specific goals of the wellness committee include: Promote a healthy work life balance. Provide physical, psychological, social and professional wellness education. Maintain a peer support and advocacy network for the residents.

The Wellness Committee, made up of attendings and residents from all years, provides resources, workshops and events to build and support the physical, psychological and emotional well-being of our emergency department.

The Department of Emergency Medicine has established a wellness committee to promote the wellness of its residents through a multifaceted approach that includes education, social programming, mentorship, and organization-directed interventions.

### Theme 5: Wellness Culture and Environment

The clinical environment can be an impediment to resident wellness. Some programs discussed their wellness culture and how they can make changes in the clinical and learning environments to positively impact their team.

Implementing projects designed to improve the meaning residents find in their daily work.

Advocating for changes in the learning environment that will improve resident well-being without compromising patient care or education.”

### Theme 6: Wellness Curriculum

Nearly all programs have a didactics section on their website, but 51.8% of programs with wellness pages featured ways that they incorporate wellness topics into their didactic sessions.

Developing a wellness curriculum that includes traditional lectures (depression, substance use), faculty panels (sleep, work-life balance), guest speakers (financial health), and experiential exercises (yoga, mindfulness).

### Theme 7: Work-Life Integration

The scheduling demands of residency are one of the drivers of decreased wellness. Many programs mentioned how their schedule and other residency requirements directly affects wellness.

Sleep loss has negative effects including learning and cognition which is why it is important to avoid sleepless nights and to watch for circadian violations.

Resident centric scheduling, maximizing vacation preferences.

Every block, each residency class will have a protected Wednesday evening as a class after Grand Rounds to spend time as a class, have social events sponsored by the residency program, catch up on appointments, errands, or have family time.

In order to decrease physician burn-out, our shifts are 8-hours in length. We also encourage our providers to stop seeing new patients 1-hour before shifts end in order to decrease charting-time past your shift. Moreover, as you progress in your residency, the total number of your shifts per block gradually decreases, allowing for more time for other activities.

### Theme 8: Growth and Development

Professional growth and development is a natural and necessary part of residency. Many programs outlined various curricula and mentoring programs that help their residents succeed professionally and improve their mental wellbeing.

The focus of coaching is to improve current performance by helping a person learn how to do things better to reach their desired outcome. The goal of coaching is to help trainees reach their peak potential, personally and professionally while in training.

Faculty mentors are chosen for their professional and life experience and ability to model and mentor healthy life/work balance and continued joy and success in their practice of medicine. Through the faculty mentorship program, residents are guided through their residency and are able to learn and adopt skills from their mentor’s years of experience.

### Theme 9: Community Involvement

Although community involvement appeared less frequently than many other themes, some programs highlighted their involvement in wellness and advocacy on a national level.

Our section strives to provide local, regional and national leadership toward improving the health and wellness of all physicians and healthcare providers. Leaders in our department have been involved in advocating for and promoting local, regional, and national change in the healthcare system with the goal to improve wellness for physicians and healthcare providers.

Forty-five programs linked to a general GME wellness page that applied to all residencies, not just EM. These pages were analyzed as well using the same codebook developed for EM pages. The most common subjects on GME wellness pages included wellness resources, mental health, physical health, and institutional support. All subjects discussed on GME pages can be found in Appendix B.

Most programs (60.79%) discussed their program’s commitment and approach to wellness on areas of their website other than a dedicated wellness page. The most common website pages on which themes of wellness were mentioned included PD/Chair Welcome, Curriculum, Why Us, Mission/Values, FAQs, and Overview. The complete list of page titles that include themes of wellness are outlined in Appendix C.

## DISCUSSION

Residency applicants strongly consider wellness when determining which programs to apply to and rank.^9^ Residency websites are one of the few ways that applicants can learn about a program’s approach to wellness. Despite this, only 68.35% of EM residency websites discussed wellness directly on their websites, and only 20.50% had website pages dedicated to wellness. Over half of programs that had wellness pages on their websites discussed social events, mental health, physical health, having institutional structure for wellness, wellness didactics, and burnout.

Forty-five programs linked to institutional pages. These pages were evaluated as well, but the topics that occurred most were different from the sub-themes on EM-specific pages. Conveying wellness information is clearest with a page dedicated to wellness, but wellness information appears throughout residency websites. Students may not access all parts of the website; so featuring wellness information on a prominent section such as a “PD Welcome” section or “Program Highlights” would be most visible.

To our knowledge, this is the first study in EM to explore residency websites for wellness-related statements. In 2021 Pollock et al performed a descriptive analysis of EM residency websites to characterize the presence of 38 items organized into the following categories: general program information; application process; research; facility information; resident information; lifestyle; and social media.^10^ Wellness was not directly assessed in their analysis. A radiology group previously performed a deductive analysis of radiology residency websites to determine the presence or absence of 26 predefined criteria related to resident wellness.^5^ They found that financial, clinical, and technical aspects of programs were commonly present on websites, but less than 10% of radiology programs mentioned resident mentoring, wellness committees, or their non-clinical curricula.

Similarly, in internal medicine a group reviewed 579 internal medicine websites for variables that a focus group found to be important to wellness and found that 81% of internal medicine websites mentioned wellness, and 41% had a page dedicated to wellness.^11^ Pavuluri et al accessed urology residency websites to determine whether the words “wellness” or “wellbeing” were used anywhere on the website and found that only 20% of programs mentioned one of these terms.^12^ Using a two-proportion z-test (*P* <0.001), we found that in EM, a significantly higher percentage of programs mentioned wellness directly.

In contrast to the reviews published in the radiology, internal medicine, and urology literature, we performed an inductive conceptual analysis. We sought to characterize all concepts discussed on residency wellness pages rather than a predetermined list of criteria. While a deductive approach employed by other groups may be less prone to bias, it also misses important content, and the depth of analysis is limited. Our approach allowed us to assess the topics that residency programs deemed important to convey to applicants or the public. Social events were the most common sub-theme on EM residency websites, with 83.9% of programs discussing events that they hold for residents. In programs that linked to a GME page highlighting wellness the most common subtheme was institutional resources.

While there are no published studies that establish a correlation between a residency website’s representation of wellness and actual resident wellbeing, describing website representation of wellness among EM programs is still valuable for residency program leadership, marketing teams, web design teams, and social media teams. We hope that this article will lead programs to enhance the quality of wellness information on their website and, more so, to continue to improve wellness initiatives for their program. After reviewing 278 EM websites, we believe that the following information should be included on EM residency program pages:

1. A subpage dedicated to wellness
2. Feature wellness information in a prominent location such as the program director’s message or the program highlights
3. The program’s approach to social activities, psychological wellbeing, health, wellness resources, culture, and curriculum focused on wellness
4. Specific examples of programming, curricula, committees, resources, or social events that increase wellness and mitigate burnout through inclusion of descriptions, photos, videos, or linked pages

A comprehensive and mission-aligned description of wellness on the program website could increase medical student interest, engagement, and ultimately recruitment to programs. Additionally, reviewing the approach to each of the themes discussed above may lead programs to improve aspects of their program’s overall wellness structure.

## LIMITATIONS

Our study provides insight into how EM residency programs convey their wellness structure and culture to applicants through their websites, but it is important to acknowledge some limitations. First, we accessed websites in Spring 2023. Because program websites are constantly evolving, the current website content and page structure may be different from when we reviewed. Additionally, websites are only one way that programs communicate information to applicants. In addition to their website, programs may use social media, virtual meet-and-greet sessions, second-look events, interview days, and other means to highlight aspects of their program’s wellness efforts. None of those communication platforms were considered in the current study.

Second, we had a small team, and coding was performed by two individuals. While we followed rigorous methodology and iteratively developed and refined a codebook, this type of analysis lends itself to bias. Third, some programs have multiple websites. In this instance, we did consider all wellness statements if they appeared on any website that was found. While our best efforts were made to include data points from any program website, it is possible we may have missed programs who have multiple websites under varying names or nicknames for the program. Finally, our content analysis was based on the presence of concepts and did not assess the detail or quality of information provided.

## CONCLUSION

Residency websites are an important resource for medical students when they are reviewing programs for residency. Information about wellness is important to most students. There is significant variation in how programs address wellness topics on their website; 75.18% of programs discuss wellness either on a dedicated wellness page, in other locations on their website, or on a linked institutional wellness page. Of the 20.5% of programs that have a dedicated page to wellness, they explore themes related to community involvement, growth and development, nutrition and health, psychological wellbeing, social and relaxation activities, wellness culture and environment, wellness curriculum, wellness structure and resources, and work-life integration. We hope this study encourages improvement in the way EM residency programs present wellness information on their websites, as the internet will continue to be a vital source of information for applicants. Future research could explore the alignment or misalignment between wellness programs offered and the perceived needs of EM residents.

## Supplementary Information



## Figures and Tables

**Table 1:** Distribution of wellness topics on emergency medicine residency wellness webpages.

Wellness topic	N (%)
Social events	47 (83.93%)
Mental health	32 (57.14%)
Physical health	31 (55.36%)
Institutional structure	30 (53.57%)
Didactics	29 (51.79%)
Burnout	28 (50.00%)
Food	27 (48.21%)
Resident wellness committee	24 (42.86%)
Culture	23 (41.07%)
Resilience and coping	23 (41.07%)
Work-life balance	22 (39.29%)
Stress	19 (33.93%)
Resources	18 (32.14%)
Finance	17 (30.36%)
Peer support	17 (30.36%)
Professional development	17 (30.36%)
Local amenities	16 (28.57%)
Mentorship	16 (28.57%)
Counseling services	13 (23.21%)
Relaxation	13 (23.21%)
Depression/suicide	11 (19.64%)
Medical health services	10 (17.86%)
Destructive habits	9 (16.07%)
Professional satisfaction	9 (16.07%)
Spiritual health	9 (16.07%)
Family and childcare	9 (16.07%)
National involvement	8 (14.29%)
Self-monitoring	8 (14.29%)
Community service	7 (12.50%)
Definition	7 (12.50%)
Schedule	7 (12.50%)
Coaching	6 (10.71%)
Efficiency	6 (10.71%)
ACGME requirements	5 (8.93%)
Advocacy	4 (7.14%)
Contract negotiations	4 (7.14%)
Scholarship	4 (7.14%)
Harassment	2 (3.57%)
Imposter syndrome	2 (3.57%)
Lack of professional fulfillment	2 (3.57%)
Leadership skill development	2 (3.57%)
Legal concerns	2 (3.57%)
Personal development	2 (3.57%)
Achievement	1 (1.79%)
Empathy	1 (1.79%)

*ACGME*, Accreditation Council for Graduate Medical Education.
