# Supplementary material for: Emergency Medicine Residency Website Wellness Pages: A Content Analysis
Source: West J Emerg Med. 2025 May 16;26(3):573–9. doi: 10.5811/westjem.34873 (PMC12208029; doi:10.5811/westjem.34873)
Supplement: Supplementary file 1 [file wjem-26-573-s001.docx]

**Appendix A.** Page titles of emergency medicine wellness pages on emergency medicine residency websites.

| **Page title** | **N (%)** |
| --- | --- |
| Wellness | 22 (38.60%) |
| Resident Wellness | 12 (21.05%) |
| Residency Wellness | 2 (3.51%) |
| Resident Life & Wellbeing | 2 (3.51%) |
| Resident Well-being | 2 (3.51%) |
| Wellness Committee | 2 (3.51%) |
| Emergency Medicine Residency Wellness | 1 (1.75%) |
| Emergency Medicine Well-being Committee | 1 (1.75%) |
| Residency Wellness & Support | 1 (1.75%) |
| Resident Wellbeing/Resilience | 1 (1.75%) |
| Resident Wellness and Social Events | 1 (1.75%) |
| Resiliency & Wellness | 1 (1.75%) |
| Well-being | 1 (1.75%) |
| Well-being, Interest Groups, and Community Service | 1 (1.75%) |
| Wellness and Wellbeing | 1 (1.75%) |
| Wellness for residents | 1 (1.75%) |
| Wellness Initiatives | 1 (1.75%) |
| Wellness Program | 1 (1.75%) |
| Wellness Resources | 1 (1.75%) |
| Wellness, Equity, Diversity, and Inclusion | 1 (1.75%) |
| Wellness, Truly | 1 (1.75%) |

**Appendix B.** Distribution of wellness topics on institutional graduate medical education wellness webpages.

| **Wellness Topic** | **N(%)** |
| --- | --- |
| Resources | 31 (68.89%) |
| Mental health | 29 (64.44%) |
| Physical health | 28 (62.22%) |
| Institutional structure | 22 (48.89%) |
| Stress | 20 (44.44%) |
| Burnout | 18 (40.00%) |
| Resilience and coping | 17 (37.78%) |
| Counseling services | 16 (35.56%) |
| Social events | 14 (31.11%) |
| Family and childcare | 13 (28.89%) |
| Culture | 12 (26.67%) |
| Finance | 11 (24.44%) |
| Professional satisfaction | 10 (22.22%) |
| Resident wellness committee | 9 (20.00%) |
| Depression/suicide | 8 (17.78%) |
| Spiritual health | 8 (17.78%) |
| Didactics | 7 (15.56%) |
| Peer support | 7 (15.56%) |
| Professional development | 7 (15.56%) |
| Self-monitoring | 7 (15.56%) |
| Medical health services | 6 (13.33%) |
| Work-life balance | 6 (13.33%) |
| ACGME requirements | 5 (11.11%) |
| Coaching | 5 (11.11%) |
| Destructive habits | 5 (11.11%) |
| Food | 5 (11.11%) |
| Local amenities | 5 (11.11%) |
| Leadership skill development | 4 (8.89%) |
| Advocacy | 3 (6.67%) |
| Community service | 3 (6.67%) |
| Efficiency | 3 (6.67%) |
| Legal concerns | 3 (6.67%) |
| Personal development | 3 (6.67%) |
| Relaxation | 3 (6.67%) |
| Definition | 2 (4.44%) |
| Harassment | 2 (4.44%) |
| Lack of professional fulfillment | 2 (4.44%) |
| Schedule | 2 (4.44%) |
| Scholarship | 2 (4.44%) |
| Empathy | 1 (2.22%) |
| IMG support | 1 (2.22%) |
| Imposter syndrome | 1 (2.22%) |

*ACGME,* Accreditation Council for Graduate Medical Education; *IMG,* international medical graduate.

**Appendix C.** Distribution of webpage titles in programs that discussed wellness on their website in areas other than a dedicated wellness page.

| **Webpage Title** | **N(%)** |
| --- | --- |
| PD Welcome/Chair Welcome | 48 (28.40%) |
| Curriculum/Longitudinal Experiences | 25 (14.79%) |
| Why us? / Highlights / Facts | 22 (13.02%) |
| Mission/Vision/Philosophy/Objectives/Aims/Values | 18 (10.65%) |
| FAQ | 18 (10.65%) |
| Overview/Homepage | 17 (10.06%) |
| Benefits | 16 (9.47%) |
| Didactics/Conferences | 15 (8.88%) |
| About/Program Info | 14 (8.28%) |
| Resident Life | 10 (5.92%) |
| DEI | 8 (4.73%) |
| Teaching/Professional Development | 8 (4.73%) |
| Orientation/Retreats/Activities | 6 (3.55%) |
| Faculty | 5 (2.96%) |
| Meet the Residents | 5 (2.96%) |
| Women in EM | 4 (2.37%) |
| Testimonials | 4 (2.37%) |
| Resources | 2 (1.18%) |
| Rotations | 2 (1.18%) |
| Life in ___ | 2 (1.18%) |
| Blog | 2 (1.18%) |
| Resident Manual | 2 (1.18%) |
| Wellness Fellowship | 1 (0.59%) |
| Research | 1 (0.59%) |
| Mentorship | 1 (0.59%) |

*PD,* Program Director; *FAQ,* frequently asked questions; *DEI,* diversity, equity, and inclusion.
